# Microstate Dynamics of Focused Attention Meditation

**DOI:** 10.1007/s10548-026-01199-2

**Published:** 2026-04-24

**Authors:** Chuong Ngo, Erkin Bek, Monika Stasytytė, Lionel A. Newman, Rodrigo Elizalde, Amit Kanthi, NK Manjunath, Christoph M. Michel

**Affiliations:** 1 All Here SA, Geneva, Switzerland; 2 Laboratory of Cognitive Neuroscience, Swiss Federal Institute of Technology Lausanne, Geneva, Switzerland; 3 Divison of Life Sciences, Swami Vivekananda Yoga Anusandhana Samsthana, Bengaluru, India; 4 Department of Basic Neurosciences, University of Geneva, Geneva, Switzerland

**Keywords:** EEG microstates, Focused attention meditation, Large-scale brain dynamics, Neural correlates of attention

## Abstract

Focused-attention meditation provides a tractable model for examining how large-scale brain dynamics support attention and self-regulation. Using high-density EEG microstate analysis, we investigated how focused-attention meditation on the breath (Ānāpānasati) modulates intrinsic brain activity in 22 experienced practitioners, compared with baseline rest and deliberate mental imagery. Five canonical microstate classes (A-E) were identified. Meditation produced a robust reduction of Microstate C across coverage, duration, and occurrence, accompanied by increased presence of Microstates D and E (all Microstate x Condition interactions p < 0.0001). Source localization revealed that Microstate C was generated primarily in medial and lateral temporal regions including the hippocampus and parahippocampal cortex, whereas Microstate D involved posterior midline regions including the posterior cingulate cortex and precuneus, and Microstate E engaged frontoparietal and orbitolimbic networks. Together, these results indicate that focused-attention meditation reorganizes the temporal architecture of large-scale brain dynamics by downregulating microstate patterns associated with self-referential and memory-based processing while enhancing neural states supporting attentional stability and internal monitoring.

**Supplementary Information:**

The online version contains supplementary material available at 10.1007/s10548-026-01199-2.

## Introduction

Meditation offers a unique window into the dynamics of human consciousness, providing a stable and trainable framework for investigating how attention, self-awareness, and mental silence emerge from large-scale brain activity (Lutz et al. 2008; Dahl et al. 2015). While numerous neuroimaging studies have shown that meditation influences oscillatory rhythms, functional connectivity, and activity within the default mode network (DMN) (Fox et al. 2016), these approaches often lack the temporal precision needed to capture the rapid transitions that shape moment-to-moment experience. EEG microstates—transient, quasi-stable topographies lasting approximately 40–120 ms—provide an ideal method for tracking such fast-changing cognitive dynamics (Lehmann et al. 1987; Michel and Koenig 2018). Conceptualized as the “atoms of thought,” microstates reflect fundamental building blocks of large-scale neural network activity and offers a dynamic tool to investigate underlying characteristics of human consciousness during rest (Custo et al. 2017; Zanesco 2024) or during altered states of consciousness (Bréchet and Michel 2022). Because meditative practices deliberately modulate *conscious states* — reducing self-related thoughts and mind-wandering while cultivating present-moment awareness (Fell et al. 2010; Lutz et al. 2015; Vago and Zeidan 2016)—microstate analysis offers a powerful, mechanistically grounded approach for understanding how meditation reorganizes the temporal structure of conscious experience. Investigating meditation through the lens of microstates therefore not only illuminates the neural signatures of contemplative training but also contributes to broader models of how the brain organizes and stabilizes states of focused awareness, introspection, and mental stillness.

Building on this concept, a growing body of intervention research demonstrates that meditation training affects the brain’s intrinsic large-scale dynamics, as captured by EEG microstates. Zanesco et al. (2021a, b) reported that two three-month Shamatha retreats led to significant decreases in microstate duration and strength across the canonical classes (A–D), along with altered transition probabilities, with subjective improvements in attentiveness and serenity showing strong explanatory power. Bréchet et al. (2021) observed a reconfiguration of microstate topographies (particularly microstates C and E) and their cortical sources, involving the right insular, the superior temporal gyrus, the superior parietal lobule, and the superior frontal gyrus bilaterally in participants after six weeks of app-based breath-focused training. Likewise, in an eight-week mindfulness-based stress reduction training (MBSR) training, Zarka et al. (2024) found significant reductions in the duration, occurrence, and coverage of microstate C, with microstate C parameters moderately correlated with the mindfulness facet of non-reactivity. Long-term style-specific expertise also shapes baseline EEG: Kopal et al. (2014) reported that practitioners of insight versus calm meditation exhibit distinct coherence patterns affecting the expression of microstates A–D even at rest. Together, these intervention studies indicate that meditation training reliably alters the spatio-temporal dynamics of large-scale brain networks.

Further evidence comes from studies measuring EEG microstates **during** meditation, showing how moment-to-moment contemplative experience is reflected in dynamic neural patterns. During open-monitoring (non-reactive attention) meditation, Zarka et al. (2021) reported a state-related decrease in microstates A and C and an increase in microstate B, with microstate temporal parameters also relating to mindfulness traits. In Transcendental Meditation, Faber et al. (2017) found significantly lower occurrence of microstates A and C during the “transcending” phase compared with undirected mentation. Early work by Faber et al. (2005) demonstrated that deep meditative absorption increases the duration of microstates B–D, while Panda et al. (2016) showed that meditation increased a DMN-linked microstate, with effects scaling with years of experience. More recent MEG work by D’Andrea et al. (2024) further revealed style-specific neural signatures, with focused-attention meditation increasing visual microstate MS1, whereas open-monitoring practice enhanced executive and ventral-attention microstates MS3 and MS5. Together, these studies suggest that meditation reliably modulates the presence of microstates associated with mind-wandering and attention depending on the specific meditation style.

Motivated by these prior observations, the present study examines microstate dynamics during Focused-Attention (FA) meditation in experienced practitioners. We conducted microstate analysis on high-density EEG recordings collected at Pyramid Valley International (Bengaluru, India), a large contemplative centre rooted in the Indian meditative culture whose primary practice is sustained awareness of the natural breath, known *as Ānāpānasati (“*Mindfulness of breathing”). *Ānāpānasati* is one of the most widely described classical breath-based meditation practices, appearing prominently in early Buddhist literature such as the Ānāpānasati Sutta (MN 118; Bodhi 2015). Within modern contemplative science, mindfulness of breathing corresponds to the Focused Attention category of meditation practices (Lutz et al. 2008). More broadly, breath-focused attention is widely employed across diverse contemplative practices as a foundational method for cultivating attentional stability, functioning as a core or preparatory technique not only in Buddhist lineages (Buddhaghosa 2011; Goenka 1997) but also in Yogic concentration practices (Feuerstein 2011), Zen breath-regulation training (Aitken 1994), modern mindfulness programs including MBSR and MBCT (Kabat-Zinn 1990; Segal et al. 2013), and various contemporary meditative approaches that use breath awareness as a gateway to deeper cognitive and experiential refinement (Brown et al. 2013).

In Ānāpānasati practice, attention is maintained on the tactile sensations of the breath—typically at the nostrils or along the natural flow of inhalation and exhalation—while minimizing cognitive elaboration, imagery, and conceptual evaluation. When distractions such as spontaneous thoughts, memories, or emotional reactions arise, practitioners gently acknowledge them and return attention to the breath, strengthening both sustained attention and meta-awareness (Dunne 2015; Vago and Zeidan 2016). Over time, this repeated cycle of focusing, distraction, and reorientation leads to increasing continuity of attention and a progressive quieting of self-referential and narrative mental activity, thereby reducing mind-wandering and enhancing cognitive stability. This core mechanism of attentional training has been well documented in contemplative cognitive research and is thought to support the development of sustained concentration and cognitive control through the systematic modulation of attentional networks (Lutz et al. 2008; Hasenkamp et al. 2012). In highly experienced meditators, sustained practice of Ānāpānasati may culminate in deep absorptive states traditionally described as Jhāna (Pāli), Dhyāna (Sanskrit), or Samādhi, characterized by markedly reduced internal dialogue, heightened perceptual clarity, and profound mental stillness (Wallace 1999; Analayo 2019). These states closely parallel descriptions of meditative absorption in the *Yoga Sūtra* of Patañjali (e.g., Yoga Sūtra I.2–I.3; Patañjali, trans. 1890), which emphasize the cessation of mental fluctuations and the stabilization of awareness in its own nature.

Although the underlying neuroscientific mechanism of FA meditation has been studied for decades, a recent systematic review (Lieberman et al. 2025) highlights substantial inconsistencies in existing literature, particularly in spectral analyses. The authors emphasize that conclusions based solely on electrode-level power measurements may be misleading, as scalp signals do not reliably reflect their underlying neural generators. They therefore recommend that future research incorporate spatially informed methods—such as EEG microstate analysis and source localization techniques including low-resolution electromagnetic tomography (LORETA)—to better characterize the neural mechanisms supporting FA.

In the present study, we follow this recommendation by applying microstate analysis to high-density EEG recorded during Ānāpānasati practice. We characterize the resulting microstate topographies during FA meditation, quantify their temporal properties (occurrence, duration, coverage), and reconstruct their cortical generators using LORETA. Finally, we assess how these microstate dynamics relate to mind-wandering, attentional stability, and self-awareness, thereby providing a more mechanistic account of the neural processes underlying FA meditation.

## Methods

### Participants and Protocol

Twenty-five subjects participated in this study. Three subjects were excluded: two performed different meditation practices and one withdrew the consent after the recording. The remaining 22 subjects includes 8 male and 14 female. Subjects’ age ranged from 27 to 77 years (M = 50.6, SD = 15.4). They reported the following durations since beginning their meditation practice: 2–5 years (8 participants), 5–10 years (4 participants), 10–20 years (9 participants), and more than 20 years (4 participants). All participants practiced *Ānāpānasati* and were recruited through recommendation of the Pyramid Valley international. All subjects signed an informed consent form. The study was approved by the institutional Ethics committee of S-VYASA (RES/IEC-SVYASA/394/2025).

All participants followed the same fixed protocol. They were seated in a meditation room either on a chair or on the floor in meditation posture. The protocol consisted of three conditions, all performed with eyes closed: First, participants were instructed to sit still and relax for 3 minutes without meditating, allowing their mind to wander freely (Baseline). Second, they were instructed to visually imagine a scene from the past as vividly as possible for 3 minutes (Mental Imagery). Finally, participants performed their usual meditation practice for 20 minutes (Meditation). There was about 1-2 minutes break between the conditions.

### Data Acquisition and Data Analysis

EEG was recorded continuously using a 64-channel net with equidistant electrode positioning (ANT Neuro). Electrodes consisted of sponges soaked in saline water. Electrode impedances were maintained below 50 kΩ. Data were acquired with 5Z as the recording reference at a sampling rate of 500 Hz.

Preprocessing: EEG data were down-sampled to 250 Hz and band-pass filtered between 2-40 Hz using a zero-phase, 4th-order Butterworth filter. A 50 Hz notch filter was applied to remove line noise. The reference electrode was added as a zero-valued channel prior to re-referencing, and the data were subsequently re-referenced to the average reference. All recordings were visually inspected for artifacts. Bad electrodes were interpolated using spherical spline interpolation, and artifact periods were marked and excluded from further analysis. Finally, data were spatially smoothed using the spatial filter implemented in the Cartool software, as described in detail in Michel and Brunet (2019).

Microstate Segmentation: A modified k-means cluster analysis (Pascual-Marqui et al. 1995) was applied to the EEG of each subject across the three eyes-closed conditions (Baseline, Mental Imagery, and Meditation) using the open-source software Cartool, following the recommendations of Bagdasarov et al. (2024). The analysis consisted of two steps:

*Individual-level clustering:* The Global Field Power (GFP) peaks of each subject’s EEG across the three conditions were extracted and subjected to k-means clustering with 50 repetitions. The data were randomly split into epochs of 5 seconds covering 99.9% of the data. This resampling was repeated 45 times to produce more reliable clustering. For each epoch, the number of clusters was set to range from 4 to 12, and the optimal number for each epoch was selected using the meta-criterion—an aggregate measure of six independent criteria implemented in the Cartool software.

*Group-level clustering:* The optimal cluster maps from each of the 45 epochs of all subjects were then subjected to a second k-means clustering with 100 resampling trials and 100 repetitions. The optimal number of clusters was determined using the meta-criterion, which yielded 5 microstate classes.

Microstate Back-fitting: The optimal cluster maps resulting from the group-level clustering were back-fitted to each subject’s data across the three conditions. Using spatial correlation of GFP-normalized maps, each time point was labeled with the cluster map that showed the highest correlation (winner-takes-all labeling), with polarity ignored. Time points that showed correlations lower than 50% with any cluster map were not labeled. After back-fitting, temporal smoothing was applied (window half-size of 40 ms and Besag factor of 10; Pascual-Marqui et al. 1995). Segments shorter than 40 ms were removed by assigning the first half to the preceding segment and the second half to the following segment. The following parameters were extracted for each microstate class: mean duration (in milliseconds), coverage (percentage of time points labeled by the microstate), and occurrence (number of segments per second).

### Statistical Analysis

Statistical analysis was performed using GraphPad Prism. The three fitting parameters (duration, coverage, and occurrence) were subjected to two-way repeated measures ANOVA with microstate class and condition as within-subject factors. Post-hoc pairwise comparisons were performed using paired t-tests with correction for multiple comparisons by controlling the false discovery rate using the linear two-stage step-up procedure (Benjamini et al. (2006) implemented in GraphPad Prism. Statistical significance was set at q = 0.05.

### Source Localization

After back-fitting, all time points labeled with a given microstate class were concatenated for each subject and each condition. For source localization, only time points with correlations >80% with the corresponding cluster map were retained. The 64 electrode positions were co-registered to the MNI-152 template head using a semi-automatic procedure implemented in Cartool. Sources were modeled using a 4-shell adaptive Local Spherical Model with Anatomical Constraints (LSMAC). This head model constructs local spheres with different radii for each electrode by estimating the thickness of the scalp, skull, cerebrospinal fluid, and brain tissue under each electrode (Brunet et al. 2011). As the source model, we used the Low-Resolution Brain Electromagnetic Tomography (LORETA; Pascual-Marqui et al. 1994) distributed linear inverse solution with 6926 solution points. The results were optimized using z-scoring implemented in Cartool, which eliminates activation biases (Michel and Brunet 2019). The activity at each solution point was averaged across time points for each microstate and then averaged across participants. The group-level source distribution was thresholded to solution points above the 95th percentile of activation values, as done in previous work (Bagdasarov et al. 2022; Bréchet et al. 2019, 2021).

## Results

### Microstate Segmentation

**Fig. 1:**
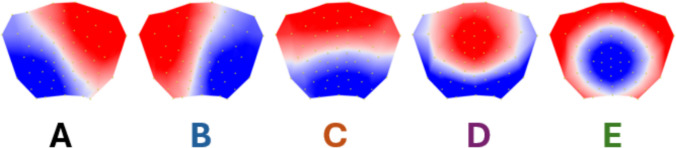
Group-level microstate segmentation identified five prototypical scalp topographies, labeled microstates A–E, as the optimal clustering solution based on a meta-criterion. The resulting maps closely resemble the canonical EEG microstate classes reported in large-scale normative studies and meta-analyses, including Koenig et al. (2024)

The meta-criterion applied to the group-level clustering identified 5 maps as the optimal number of clusters. These maps are illustrated in Fig. 1. The topographies of the five maps were highly similar to the canonical maps reported in the meta-analysis of 50 studies by Tarailis et al. (2024) and the Meta-microstates from 40 studies reported by Keonig et al., (2024) and were labeled as microstate maps A through E.

### Fitting Parameters

The fitting parameters (duration, coverage, and occurrence) were analyzed separately using two-way repeated measures ANOVAs with microstate class (5 levels) and condition (3 levels: Baseline, Mental Imagery, Meditation) as within-subject factors. The Means and standard deviations of all parameters are given in Table 1.

**Table 1:** Mean and standard deviation of all parameters

Coverage (%)										
	Map A		Map B		Map C		Map D		Map E	
	Mean	SD	Mean	SD	Mean	SD	Mean	SD	Mean	SD
Baseline	20.56186	4.642054	17.22294	4.59109	43.48598	11.76594	9.38555	6.838873	9.343655	3.873318
Imagery	18.9713	5.314025	16.72713	6.93975	35.47765	13.92304	20.1942	15.27894	8.629709	3.712457
Meditation	18.51317	3.60451	16.11599	4.995785	31.63711	10.37308	22.21672	11.72225	11.51699	3.97093
Duration (msec)										
Baseline	56.68145	3.50937	55.00335	3.442059	81.07802	15.89317	48.90991	5.648863	47.98876	3.108798
Imagery	55.74775	4.119175	54.13887	5.235733	71.31595	15.44612	57.60049	13.03384	48.19711	3.286952
Meditation	54.33019	2.73789	52.63937	3.5396	65.81837	9.552704	58.28632	10.86304	49.49987	3.032632
Occurrence(per sec)										
Baseline	3.090195	0.53714	2.689236	0.597149	3.917568	0.432858	1.603014	0.928385	1.714355	0.555199
Imagery	2.877359	0.687755	2.57495	0.855469	3.706532	0.660861	2.545673	1.261787	1.553295	0.552672
Meditation	2.921345	0.487601	2.615032	0.660491	3.705641	0.592503	2.974623	0.967565	2.016491	0.567175

### Coverage

**Fig. 2:**
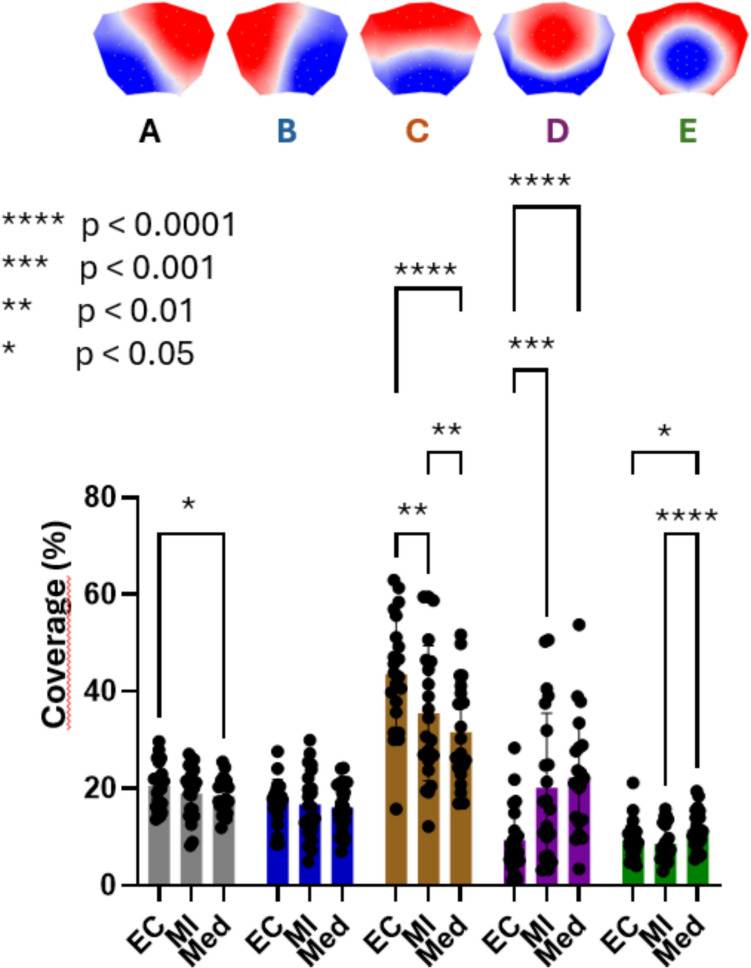
Microstate coverage across conditions. Mean coverage (%) of microstates A–E during Baseline (BL), Mental Imagery (MI), and Meditation (Med). A two-way repeated-measures ANOVA revealed a significant main effect of Microstate and a significant Microstate × Condition interaction. Overall, microstates A–C showed higher coverage than microstates D and E. Post-hoc comparisons (FDR-corrected) indicated reduced coverage of microstate C during MI and Med relative to BL, with a further reduction during Med compared to MI. In contrast, microstate D showed increased coverage during MI and Med relative to BL, with an additional increase during Med. Microstate E coverage increased during Med relative to both BL and MI, while microstate A showed a modest decrease during Med compared to BL. No significant condition effects were observed for microstate B. Significance levels are indicated in the figure. All p-values are FDR-corrected

The two-way ANOVA revealed a significant main effect of Microstate (F(1.861, 39.08) = 32.05; p < 0.0001), and a significant Microstate × Condition interaction (F(2.560, 53.76) = 17.46; p < 0.0001). The main effect of Microstate reflected generally higher coverage of microstates A, B, and C compared to microstates D and E (Fig. 2).

Post-hoc pairwise comparisons corrected for false discovery rate revealed the following pattern for the Microstate × Condition interaction: Microstate C showed significantly decreased coverage during both Mental Imagery and Meditation compared to Baseline, with a further significant decrease during Meditation compared to Mental Imagery. Conversely, microstate D coverage significantly increased during both Mental Imagery and Meditation compared to Baseline, with an additional increase during Meditation compared to Mental Imagery. Microstate E coverage increased significantly during Meditation compared to both Baseline and Mental Imagery, while no difference was observed between Baseline and Mental Imagery. Microstate A showed a modest but significant decrease in coverage during Meditation compared to Baseline. Microstate B coverage did not differ significantly across conditions.

### Duration

**Fig. 3:**
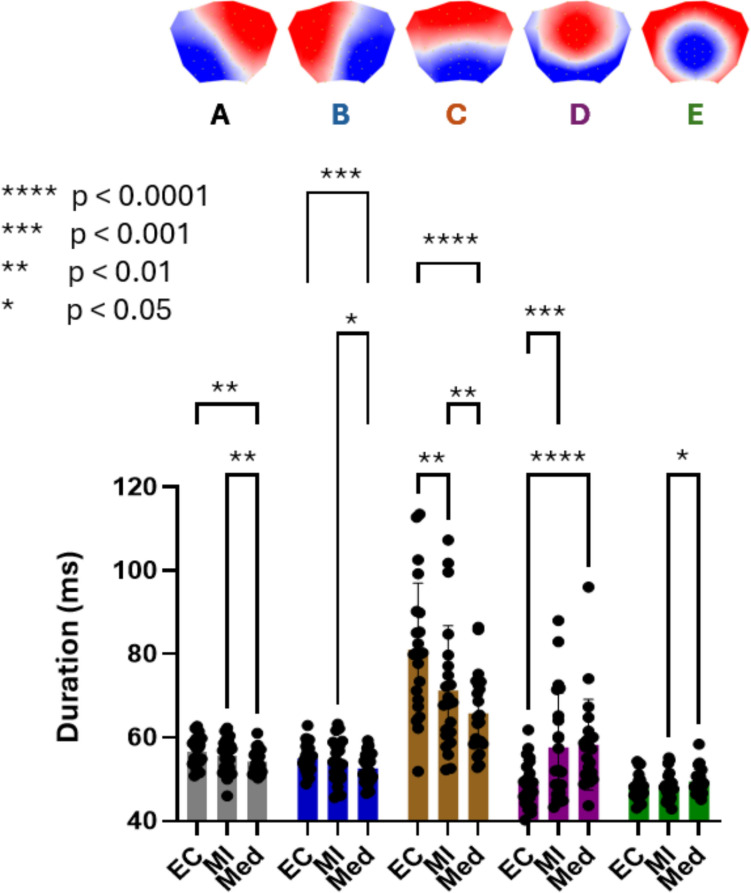
Microstate duration across conditions. Mean duration (ms) of microstates A–E across Baseline (BL), Mental Imagery (MI), and Meditation (Med). Microstate C showed longer overall durations than the other microstates. A significant Microstate × Condition interaction indicated reduced duration of microstates A–C during Meditation relative to BL and MI, increased duration of microstate D during Meditation relative to BL, and increased duration of microstate E during Meditation relative to MI. Mental Imagery relative to BL was associated with reduced duration of microstate C and increased duration of microstate E. Significance levels are indicated in the figure. All p-values are FDR-corrected

The two-way repeated measures ANOVA revealed significant main effects for both Microstate (F(1.724, 36.20) = 31.21; p < 0.0001) and Condition (F(1.554, 32.63) = 5.721; p = 0.012), as well as a significant Microstate × Condition interaction (F(2.707, 56.84) = 16.16; p < 0.0001). Microstate C showed generally longer mean duration than the other microstates across all conditions (Fig. 3).

Post-hoc comparisons revealed that during Meditation compared to Baseline, microstates A, B, and C showed decreased duration, while microstate D showed increased duration. Compared to Mental Imagery, Meditation led to decreased duration of microstates A, B, and C, and increased duration of microstate E. Mental Imagery compared to Baseline resulted in decreased duration of microstate C and increased duration of microstate E.

### Occurrence

**Fig. 4:**
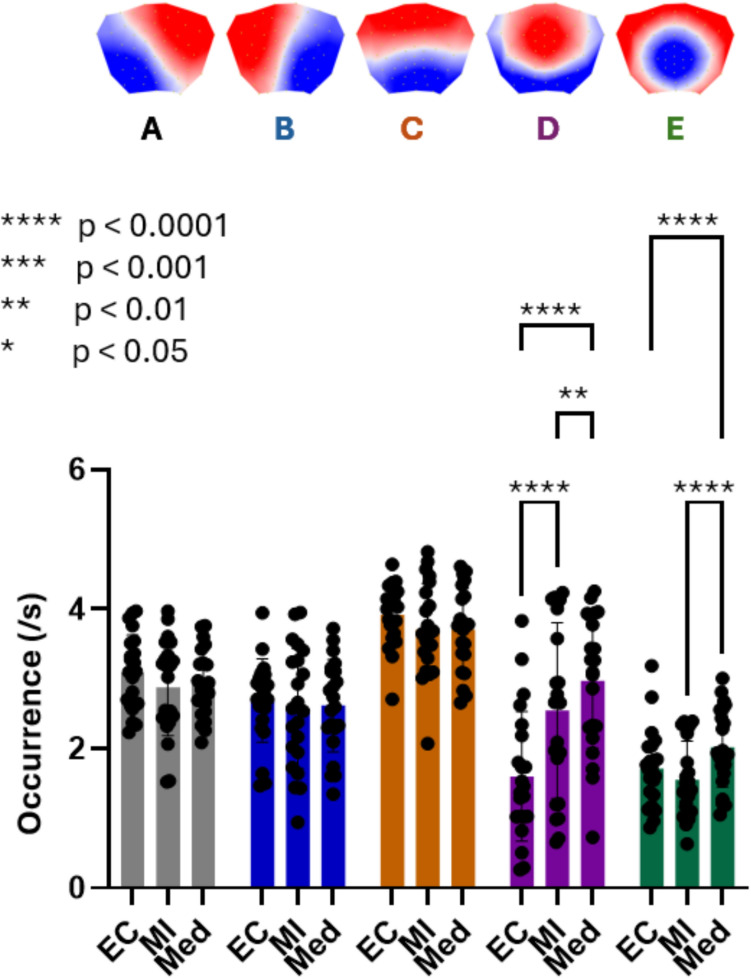
Microstate occurrence across conditions. Mean occurrence rate (events/s) of microstates A–E across Baseline (BL), Mental Imagery (MI), and Meditation (Med). A two-way repeated-measures ANOVA revealed significant main effects of Microstate and Condition, as well as a significant Microstate × Condition interaction. Post-hoc comparisons (FDR-corrected) showed increased occurrence of microstates D and E during Meditation relative to both BL and MI. Mental Imagery was also associated with increased occurrence of microstate D compared to BL. Significance levels are indicated in the figure. All p-values are FDR-corrected

The two-way ANOVA revealed significant main effects for Microstate (F(1.852, 38.88) = 27.36; p < 0.0001) and Condition (F(1.612, 33.86) = 7.297; p < 0.01), as well as a significant Microstate × Condition interaction (F(3.745, 78.65) = 22.30; p < 0.0001). Post-hoc tests revealed that microstates D and E showed significantly increased occurrence during Meditation compared to both Baseline and Mental Imagery. Mental Imagery also showed significantly increased microstate D occurrence compared to Baseline (Fig. 4).

### Source Localization

**Fig. 5:**
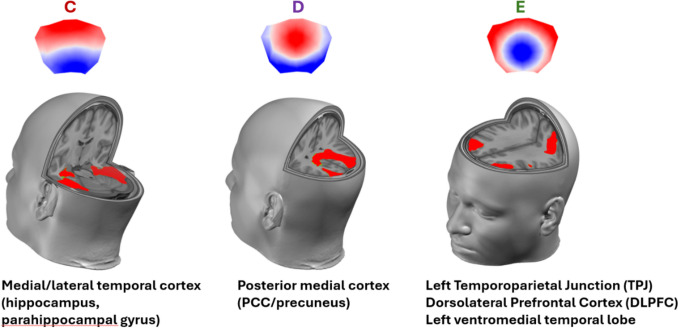
Distributed source estimates for microstates C–E, illustrating distinct cortical networks associated with each microstate class. Canonical scalp topographies are shown above the corresponding source maps

Source localization of the microstates revealed distinct networks for each microstate class (Fig. 5 and Supplementary Figure 1). Microstates A and B showed activity in the right and left temporal pole, respectively. Microstate C, which decreased during meditation, was localized in the medial/lateral temporal cortex, encompassing the hippocampus, parahippocampal gyrus, and middle temporal gyrus. Microstate D, which increased during meditation, showed main activity in the posterior medial cortex, including the posterior cingulate cortex (PCC), precuneus, and cuneus bilaterally. Microstate E, which also increased during meditation, showed three main areas of activity: the left temporoparietal junction (TPJ) including the angular gyrus and supramarginal gyrus, the dorsolateral prefrontal cortex (DLPFC) bilaterally, and the left orbitolimbic region including the amygdala and extending to orbital frontal cortex.

## Discussion

The present study investigated how focused-attention (FA) meditation on the breath modulates the temporal dynamics and neural generators of EEG microstates in experienced practitioners. Across multiple metrics—coverage, duration, and occurrence—we observed a consistent reorganization of microstate dynamics during meditation relative to both baseline rest and deliberate mental imagery. These findings extend the few prior work showing that meditation modulates the dynamics of large-scale neural networks measured with EEG microstates (Zarka et al. 2021; Faber et al. 2005, 2017; Panda et al. 2016).

Among all microstates, Microstate C emerged as the dominant temporal state during baseline, showing the longest mean duration as well as the highest occurrence and coverage. Relative to baseline, Microstate C was significantly reduced during both Mental Imagery and Meditation, with the strongest attenuation observed in the Meditation condition.

The effect of focused-attention (FA) meditation on Microstate C is comparable to that observed during non-autobiographical, externally oriented tasks such as mental arithmetic. Bréchet et al. (2019) reported reduced duration and occurrence of Microstate C during mental arithmetic compared with no-task rest and autobiographical memory tasks. Other studies have similarly shown longer Microstate C durations during unconstrained rest relative to arithmetic tasks (Kim et al. 2021; Seitzman et al. 2017). Croce et al. (2018), using magnetic stimulation, also linked Microstate C to task-negative mentation—mind-wandering, self-related thoughts, and emotional or interoceptive processing. Further support comes from studies examining prestimulus microstate dynamics during sustained attention tasks: Zanesco et al. (2021a, b) found that microstate C was specifically associated with episodes of self-reported mind wandering, whereas microstate E was predominant during on-task focused attention. Di Muccio et al. (2023) similarly reported that microstate C predicted slower overall reaction times, while microstates D and E predicted faster reaction times. These findings directly corroborate our interpretation of reduced microstate C during meditation as reflecting a suppression of spontaneous mind wandering and self-referential thought.

Source localization revealed that Microstate C was generated primarily within the hippocampus, parahippocampal gyrus, and middle temporal gyrus—regions strongly implicated in episodic memory retrieval and autobiographical processing during mind wandering (Faber and Mills 2018; Christoff et al. 2016; Andrews-Hanna 2012). This spatial profile aligns with prior evidence identifying Microstate C as being associated with autobiographical mentation (Tarailis et al. 2024) and are consistent with earlier work showing Microstate C sources in parietal and temporal brain regions, including the hippocampus (Bréchet et al. 2019, 2021; Tarailis et al. 2025). The suppression of Microstate C during meditation therefore likely reflects reduced engagement of hippocampal–temporal memory systems, a sub-network of the default mode network (DMN) (Andrews-Hanna 2012), and a downregulation of spontaneous self-related thoughts and autobiographical mind-wandering. Within the framework of FA meditation, this reduction may index the practitioner’s capacity to detect the emergence of self-referential distractions and reorient attention toward the meditation object.

Microstates D and E showed complementary increases during meditation, reflecting a coordinated engagement of attentional control and self-regulatory systems. Microstate D, which increased in coverage, occurrence, and duration across both Mental Imagery and Meditation, was generated primarily in the posterior medial cortex, including the posterior cingulate cortex (PCC), precuneus, and cuneus, This network functions as a critical node for modulating attention between internal self-referential processing and external task demands (Leech and Sharp 2014; Raichle et al. 2001). The PCC shows characteristic deactivation during externally focused, attentionally demanding tasks, while increased activity supports internally directed cognition (Leech et al. 2012), reflecting its role as a regulatory switch for attentional orientation (Leech et al. 2011).Previous studies have similarly localized Microstate D to parietal regions and have linked it to working memory, cognitive control, attentional reorientation, and the detection of behaviorally relevant stimuli (Croce et al. 2018; Tarailis et al. 2024). Increased presence of Microstate D has also been observed during arithmetic and recognition tasks compared with no-task rest (Bréchet et al. 2019; Kim et al. 2021; D’Croz-Baron et al. 2021; Murphy et al. 2018; Ferat et al., 2025). Additional work has associated greater Microstate D presence with heightened alertness to mental processing (Faber et al. 2017) and with transitions between internally and externally oriented cognition (Milz et al. 2016).

In the context of our study on focused-attention meditation, the increased presence of Microstate D suggests a mechanism by which the posterior medial cortex supports maintaining the internal task object (e.g., the breath at the nostril) and facilitates redirecting attention when distraction occurs. This pattern indicates that Microstate D reflects the establishment of a coherent internal attentional mode that becomes more prominent as participants transition from baseline into meditative states. This interpretation is further supported by Di Muccio et al. (2023), who reported that microstate D predicted faster reaction times during a sustained attention task, linking it to attentional engagement and efficient cognitive processing. The enhancement of Microstate D during meditation also aligns with fMRI findings demonstrating the PCC’s involvement in sustaining internal focus during meditative states (Kral et al. 2019).

Microstate E, which showed selective increases during Meditation, was localized to left temporoparietal junction (TPJ), the dorsolateral prefrontal cortex, and orbitolimbic areas including the amygdala. This three-component network provides a comprehensive neural substrate for meditative self-awareness. The TPJ is specifically implicated in self-other distinction processes and bodily self-awareness (Ionta et al. 2011; Blanke et al. 2015; Igelström and Graziano 2017; Bréchet et al. 2018) critical for the sense of self during meditation. The DLPFC has been implicated in metacognitive self-awareness through both neuroimaging and brain stimulation studies (Fleming and Dolan 2012; Lapate et al., 2020). Finally, the orbitolimbic region including amygdala and orbitofrontal cortex is associated with emotional self-awareness in meditation practitioners, with structural and functional changes in these regions correlating with mindfulness traits (Murakami et al. 2012; Baltruschat et al. 2021). This integrated network thus captures cognitive (DLPFC), perceptual (TPJ), and affective (orbitolimbic) dimensions of self-awareness characteristic of meditative states. Notably, the increase in microstate E during meditation is consistent with findings from sustained attention paradigms, where microstate E was associated with on-task focused attention relative to mind wandering (Zanesco et al. 2021a, b) and with faster reaction times (Di Muccio et al. 2023). While those studies emphasize an attentional interpretation, focused attention and self-awareness are not dissociable in FA meditation: sustaining awareness of the breath necessarily involves continuous monitoring of one’s own mental state, suggesting that microstate E may reflect a neural signature common to both attentional focus and introspective self-awareness.

The findings also raise important questions for future research—particularly concerning the specific functional contributions of the DMN and the PCC during meditation. Although meditative training is often associated with reductions in DMN activity including the PCC (Brewer et al. 2011); Garrison et al. (2015) to diminish internal narrative processes and mind-wandering, recent work indicates that the DMN is not a uniform network but a constellation of interacting subsystems with potentially divergent roles. Prior studies have identified at least two major DMN subsystems—a medial temporal subsystem involved in memory-based simulation, and a dorsal medial subsystem linked to social-cognitive and conceptual processing—along with midline hubs such as the anterior medial prefrontal cortex and the PCC that integrate information across these components. These subsystems collectively support internally oriented cognition, including spontaneous thought, mental time travel, and reasoning about the self and others (Buckner et al. 2008; Andrews-Hanna et al. 2014). Our source localization results suggest that these DMN subsystems may be differentially reflected in distinct microstates: Microstate C corresponds to the medial temporal subsystem (hippocampus, parahippocampal cortex), Microstate D appears linked to PCC activity, and Microstate E aligns with regions associated with the dorsal medial subsystem, including the TPJ. This mapping implies that meditation may reorganize interactions *within* the DMN rather than simply suppressing it globally. This interpretation aligns well with the observation of Panda et al. (2016) in a simultaneous EEG-fMRI study where meditation decreased the connectivity of the PCC in the fMRI but increased the activity of the PCC-related microstate. This discrepancy highlights an important direction for future research: examining how the temporal properties of Microstate D relate to the stabilization of attention and determining whether its source localization remains anchored in the PCC across different attentional states. Such work could illuminate the nuanced interplay between DMN subsystems and attentional regulation during meditation.

## Limitations

Several limitations of the present study should be acknowledged. First, the relatively small sample size warrants caution, particularly regarding the source localization results, whose stability and spatial specificity will benefit from confirmation in larger independent cohorts. We note, however, that small sample sizes are a recognized constraint across the field when studying experienced meditators: recent MEG work by D’Andrea et al. (2024) and Pascarella et al. (2025) included only 10 and 12 experienced monks, respectively. Our sample of 25 participants is comparably experienced, with the majority having practiced for more than 10 years (13 out of 25), all practicing Ānāpānasati, and all recruited through the Pyramid Valley International network, reflecting a dedicated practitioner population. Data collection is ongoing, and a larger dataset spanning a wider range of experience levels and traditions will allow us to assess the replicability of the present findings in future work.

Second, source localization results should be interpreted with caution, as they are sensitive to methodological choices including the number of microstate classes, the inverse solution employed, and the strategy used to link microstate assignment to source activity. The source localizations reported here differ in part from those of Custo et al. (2017), where a different number of microstates and a two-step GLM approach were used. However, the localizations obtained with our current approach are consistent with those reported in more recent studies using comparable methods (Bréchet et al. 2019; 2021; Tarailis et al. 2024; Tarailis et al. 2025), lending confidence particularly to the source localization of microstate C.

Finally, while the present findings characterize the large-scale network-level correlates of focused-attention meditation, the biological mechanisms underlying the observed reorganization of microstate dynamics remain to be fully elucidated. EEG microstate analysis captures the spatiotemporal dynamics of large-scale cortical network configurations but does not directly reveal the underlying cellular or circuit-level processes. Identifying how meditation drives these changes — whether through sustained top-down attentional modulation, changes in thalamocortical gating, or shifts in neuromodulatory tone — will require converging evidence from multimodal approaches combining EEG with fMRI, autonomic measures, or pharmacological manipulations. We consider the present findings as a characterization of the network-level correlates of meditative states, and acknowledge that the mechanistic question remains open.

## Conclusion

Focused-attention meditation reliably reorganized EEG microstate dynamics. Compared with baseline rest and mental imagery, meditation produced a strong reduction of Microstate C (coverage, duration, occurrence) alongside increased Microstates D and E. Source localization suggests a shift away from medial/lateral temporal generators (hippocampus–parahippocampal regions; memory/self-generated mentation) toward posterior midline and frontoparietal/orbitolimbic networks supporting attentional stability, internal monitoring, and self-awareness. These results indicate that FA meditation reshapes the millisecond-scale temporal architecture of large-scale brain states.

## Supplementary Information

Below is the link to the electronic supplementary material.

Supplementary file1 (DOCX 552 KB)

## Data Availability

No datasets were generated or analysed during the current study.

## References

[CR1] Aitken R (1994) The practice of Zen. HarperCollins

[CR2] Anālayo B (2019) Mindfulness in early Buddhism: Characteristics and functions. Windhorse Publications

[CR3] Andrews-Hanna JR (2012) The brain’s default network and its adaptive role in internal mentation. Neurosci 18(3):251–270. 10.1177/1073858411403316 10.1177/1073858411403316 PMC3553600 21677128

[CR4] Andrews-Hanna JR, Smallwood J, Spreng RN (2014) The default network and self-generated thought: Component processes, dynamic control, and clinical relevance. Ann N Y Acad Sci 1316(1):29–52. 10.1111/nyas.12360 24502540 10.1111/nyas.12360 PMC4039623

[CR5] Bagdasarov A, Roberts K, Bréchet L, Brunet D, Michel CM, Gaffrey MS (2022) Spatiotemporal dynamics of EEG microstates in four- to eight-year-old children: Age- and sex-related effects. Dev Cogn Neurosci 57:101134 35863172 10.1016/j.dcn.2022.101134 PMC9301511

[CR6] Bagdasarov A, Brunet D, Michel CM, Gaffrey MS (2024) Microstate analysis of continuous infant EEG: Tutorial and reliability. Brain Topogr 37:496–513. 10.1007/s10548-024-01043-5 38430283 10.1007/s10548-024-01043-5 PMC11199263

[CR7] Baltruschat S, Cándido A, Maldonado A, Verdejo-Lucas C (2021) There is more to mindfulness than emotion regulation: A study on brain structural networks. Front Psychol 12:659403. 10.3389/fpsyg.2021.659403 33868133 10.3389/fpsyg.2021.659403 PMC8046916

[CR8] Benjamini Y, Krieger AM, Yekutieli D (2006) Adaptive linear step-up procedures that control the false discovery rate. Biometrika 93(3):491–507

[CR9] Blanke O, Slater M, Serino A (2015) Behavioral, neural, and computational principles of bodily self-consciousness. Neuron 88:145–166. 10.1016/j.neuron.2015.09.029 26447578 10.1016/j.neuron.2015.09.029

[CR10] Bodhi B (2015) The middle length discourses of the Buddha: A translation of the Majjhima Nikāya. Wisdom Publications

[CR11] Bréchet L, Grivaz P, Gauthier B, Blanke O (2018) Common recruitment of angular gyrus in episodic autobiographical memory and bodily self-consciousness. Front Behav Neurosci 12:270. 10.3389/fnbeh.2018.00270 30487740 10.3389/fnbeh.2018.00270 PMC6246737

[CR12] Bréchet L, Brunet D, Birot G, Gruetter R, Michel CM, Jorge J (2019) Capturing the spatiotemporal dynamics of self-generated, task-initiated thoughts with EEG and fMRI. Neuroimage 194:82–92. 10.1016/j.neuroimage.2019.03.029 30902640 10.1016/j.neuroimage.2019.03.029

[CR13] Bréchet L, Ziegler DA, Simon AJ, Brunet D, Gazzaley A, Michel CM (2021) Reconfiguration of electroencephalography microstate networks after breath-focused, digital meditation training. Brain Connectivity 11(2):146–155. 10.1089/brain.2020.0818 33403921 10.1089/brain.2020.0848 PMC7984939

[CR14] Bréchet L, Michel CM (2022) EEG microstates in altered states of consciousness. Front Psychol 13:856697 35572333 10.3389/fpsyg.2022.856697 PMC9094618

[CR15] Brewer JA, Worhunsky PD, Gray JR, Tang Y-Y, Weber J, Kober H (2011) Meditation experience is associated with differences in default mode network activity and connectivity. Proc Natl Acad Sci 108(50):20254–20259. 10.1073/pnas.1112029108 22114193 10.1073/pnas.1112029108 PMC3250176

[CR16] Brown KW, Creswell JD, Ryan RM (eds) (2013) Handbook of mindfulness. Guilford Press

[CR17] Brunet D, Murray MM, Michel CM (2011) Spatiotemporal analysis of multichannel EEG: CARTOOL. Int J Biomed Imaging 2011:813870. 10.1155/2011/813870 10.1155/2011/813870 PMC3022183 21253358

[CR18] Buckner RL, Andrews-Hanna JR, Schacter DL (2008) The brain’s default network: anatomy, function, and relevance to disease. Ann N Y Acad Sci 1124:1–38. 10.1196/annals.1440.011 18400922 10.1196/annals.1440.011

[CR19] Buddhaghosa. (2011). The path of purification (Visuddhimagga) (Ñāṇamoli, Trans.). Buddhist Publication Society.

[CR20] Christoff K, Irving ZC, Fox KCR, Spreng RN, Andrews-Hanna JR (2016) Mind-wandering as spontaneous thought: a dynamic framework. Nat Rev Neurosci 17(11):718–731. 10.1038/nrn.2016.113 27654862 10.1038/nrn.2016.113

[CR21] Croce P, Zappasodi F, Capotosto P (2018) Offline stimulation of human parietal cortex differently affects resting EEG microstates. Sci Rep 8(1):1287. 10.1038/s41598-018-19698-z 29352255 10.1038/s41598-018-19698-z PMC5775423

[CR22] Custo A, Van De Ville D, Wells WM, Tomescu MI, Brunet D, Michel CM (2017) Electroencephalographic resting-state networks: microstate spectral sequences and their relation to fMRI resting-state networks. Neuroimage 146:438–451. 10.1016/j.neuroimage.2016.10.041 28938855 10.1089/brain.2016.0476 PMC5736178

[CR23] Dahl CJ, Lutz A, Davidson RJ (2015) Reconstructing and deconstructing the self: cognitive mechanisms in meditation practice. Trends Cogn Sci 19(9):515–523. 10.1016/j.tics.2015.07.001 26231761 10.1016/j.tics.2015.07.001 PMC4595910

[CR24] D’Andrea A, Croce P, O’Byrne J, Jerbi K, Pascarella A, Raffone A, Pizzella V, Marzetti L (2024) Mindfulness meditation styles differently modulate source-level MEG microstate dynamics and complexity. Front Neurosci 18:1295615. 10.3389/fnins.2024.1295615 38370436 10.3389/fnins.2024.1295615 PMC10869546

[CR25] D’Croz-Baron DF, Baker M, Michel CM, Kirov R, Wamsley EJ (2021) Auditory and visual tasks influence the temporal dynamics of EEG microstates during post-encoding rest. Neurobiol Learn Mem 177:107343 33095401 10.1007/s10548-020-00802-4

[CR26] Di Muccio F, Simonet M, Brandner C, Ruggeri P, Barral J (2023) Cardiorespiratory fitness modulates prestimulus EEG microstates during a sustained attention task. Front Neurosci 17:1188695 37397452 10.3389/fnins.2023.1188695 PMC10308046

[CR27] Dunne JD (2015) Buddhist styles of mindfulness: A heuristic approach. In: Brown KW, Creswell JD, Ryan RM (eds) Handbook of mindfulness. Guilford Press, pp 250–270

[CR28] Faber PL, Lehmann D, Barendregt H, Kaelin M, Gianotti LRR (2005) Increased duration of EEG microstates during meditation. Brain Topogr 18(2):131–139. 10.1007/s10548-005-6714-5

[CR29] Faber PL, Travis F, Milz P, Parim N, Laneres J, Lehmann D (2017) EEG microstates during different phases of Transcendental Meditation practice. Cogn Process 18(3):307–314. 10.1007/s10339-017-0808-6 28451913 10.1007/s10339-017-0812-y

[CR30] Faber M, Mills C (2018) The critical role of the hippocampus in mind wandering. J Neurosci 38(29):6439–6441. 10.1523/JNEUROSCI.0995-18.2018 30021763 10.1523/JNEUROSCI.0995-18.2018 PMC6705953

[CR31] Fell J, Axmacher N, Haupt S (2010) From alpha to gamma: electrophysiological correlates of meditation-related states of consciousness. Med Hypotheses 75(2):218–224. 10.1016/j.mehy.2010.02.025 20227193 10.1016/j.mehy.2010.02.025

[CR32] Feuerstein G (2011) The yoga tradition: Its history, literature, philosophy, and practice. Hohm Press

[CR33] Fleming SM, Dolan RJ (2012) The neural basis of metacognitive ability. Philos Trans R Soc B Biol Sci 367(1594):1338–1349. 10.1098/rstb.2011.0417 10.1098/rstb.2011.0417 PMC3318765 22492751

[CR34] Fox KCR, Dixon ML, Nijeboer S, Girn M, Floman JL, Lifshitz M, Christoff K (2016) Functional neuroanatomy of meditation: a review and meta-analysis of 78 functional neuroimaging investigations. Neurosci Biobehav Rev 65:208–228. 10.1016/j.neubiorev.2016.03.021 27032724 10.1016/j.neubiorev.2016.03.021

[CR35] Garrison KA, Zeffiro TA, Scheinost D, Constable RT, Brewer JA (2015) Meditation leads to reduced default mode network activity beyond an active task. Cogn Affect Behav Neurosci 15(3):712–720 25904238 10.3758/s13415-015-0358-3 PMC4529365

[CR36] Goenka, S. N. (1997). The art of living: Vipassana meditation as taught by S. N. Goenka. Vipassana Research Institute.

[CR37] Hasenkamp W, Wilson-Mendenhall C, Duncan E, Barsalou L (2012) Mind wandering and attention during focused meditation: a fine-grained temporal analysis of fluctuating cognitive states. Neuroimage 59(1):750–760. 10.1016/j.neuroimage.2011.07.008 21782031 10.1016/j.neuroimage.2011.07.008

[CR38] Igelström KM, Graziano MSA (2017) The inferior parietal lobule and temporoparietal junction: a network perspective. Neuropsychologia 105:70–83. 10.1016/j.neuropsychologia.2017.01.001 28057458 10.1016/j.neuropsychologia.2017.01.001

[CR39] Ionta S, Heydrich L, Lenggenhager B, Mouthon M, Fornari E, Chapuis D, Gassert R, Blanke O (2011) Multisensory mechanisms in temporo-parietal cortex support self-location and first-person perspective. Neuron 70(2):363–374. 10.1016/j.neuron.2011.03.009 21521620 10.1016/j.neuron.2011.03.009

[CR40] Kabat-Zinn J (1990) Full catastrophe living. Delta

[CR41] Kim K, Duc NT, Choi M, Lee B (2021) EEG microstate features according to performance on a mental arithmetic task. Sci Rep 11(1):343. 10.1038/s41598-020-79423-7 33431963 10.1038/s41598-020-79423-7 PMC7801706

[CR42] Koenig T, Diezig S, Kalburgi SN, Antonova E, Artoni F, Bréchet L, Britz J, Croce P, Custo A, Damborská A, Deolindo C, Heinrichs M, Kleinert T, Liang Z, Murphy MM, Nash K, Nehaniv C, Schiller B, Smailovic U, Tarailis P, Tomescu M, Toplutaş E, Vellante F, Zanesco A, Zappasodi F, Zou Q, Michel CM (2024) EEG-meta-microstates: towards a more objective use of resting-state EEG microstate findings across studies. Brain Topogr 37(2):218–231. 10.1007/s10548-023-00993-6 37515678 10.1007/s10548-023-00993-6 PMC10884358

[CR43] Kopal J, Vyšata O, Burian J, Schätz M, Procházka A, Vališ M (2014) Complex continuous wavelet coherence for EEG microstates detection in insight and calm meditation. Conscious Cogn 30:13–23. 10.1016/j.concog.2014.07.013 25129036 10.1016/j.concog.2014.07.015

[CR44] Kral TRA, Imhoff-Smith T, Dean DC, Grupe D, Adluru N, Patsenko E, Mumford JA, Goldman RR, Rosenkranz MA, Davidson RJ (2019) Mindfulness-Based Stress Reduction–related changes in posterior cingulate resting brain connectivity. Soc Cogn Affect Neurosci 14(7):777–787. 10.1093/scan/nsz050 31269203 10.1093/scan/nsz050 PMC6778831

[CR45] Leech R, Braga R, Sharp DJ (2012) Echoes of the brain within the posterior cingulate cortex. J Neurosci 32(1):215–222. 10.1523/JNEUROSCI.3689-11.2012 22219283 10.1523/JNEUROSCI.3689-11.2012 PMC6621313

[CR46] Leech R, Kamourieh S, Beckmann CF, Sharp DJ (2011) Fractionating the default mode network: distinct contributions of the ventral and dorsal posterior cingulate cortex to cognitive control. J Neurosci 31(9):3217–3224. 10.1523/JNEUROSCI.5626-10.2011 21368033 10.1523/JNEUROSCI.5626-10.2011 PMC6623935

[CR47] Leech R, Sharp DJ (2014) The role of the posterior cingulate cortex in cognition and disease. Brain 137(1):12–32. 10.1093/brain/awt162 23869106 10.1093/brain/awt162 PMC3891440

[CR48] Lehmann D, Ozaki H, Pal I (1987) EEG alpha map series: brain micro-states by space-oriented adaptive segmentation. Electroencephalogr Clin Neurophysiol 67(3):271–288 2441961 10.1016/0013-4694(87)90025-3

[CR49] Lieberman G, McConnell PA, Estarellas M, Sacchet MD (2025) Neurophysiological mechanisms of focused attention meditation: a scoping systematic review. Imaging Neurosci. 10.1162/IMAG.a.14. (**Advance online publication**) 10.1162/IMAG.a.14 PMC12327082 40800838

[CR50] Lutz A, Slagter H, Dunne JD, Davidson RJ (2008) Attention regulation and monitoring in meditation. Trends Cogn Sci 12(4):163–169. 10.1016/j.tics.2008.01.005 18329323 10.1016/j.tics.2008.01.005 PMC2693206

[CR51] Lutz A, Jha AP, Dunne JD, Saron CD (2015) Investigating the phenomenological matrix of mindfulness-related practices from a neurocognitive perspective. Am Psychol 70(7):632–658. 10.1037/a0039585 26436313 10.1037/a0039585 PMC4608430

[CR52] Michel CM, Brunet D (2019) EEG source imaging: a practical review of the analysis steps. Front Neurol 10:325. 10.3389/fneur.2019.00325 31019487 10.3389/fneur.2019.00325 PMC6458265

[CR53] Michel CM, Koenig T (2018) EEG microstates as a tool for studying the temporal dynamics of whole-brain neuronal networks: a review. Neuroimage 180:577–593 29196270 10.1016/j.neuroimage.2017.11.062

[CR54] Milz P, Faber PL, Lehmann D, Koenig T, Kochi K, Pascual-Marqui RD (2016) The functional significance of EEG microstates—associations with modalities of thinking. Neuroimage 125:643–656 26285079 10.1016/j.neuroimage.2015.08.023

[CR55] Murakami H, Nakao T, Matsunaga M, Kasuya Y, Shinoda J, Yamada J, Ohira H (2012) The structure of mindful brain. PLoS ONE 7(9):e46377. 10.1371/journal.pone.0046377 23029500 10.1371/journal.pone.0046377 PMC3460809

[CR56] Murphy M et al (2018) Recurrence of task-related electroencephalographic activity during post-training quiet rest and sleep. Sci Rep 8:5398. 10.1038/s41598-018-23590-1 29599462 10.1038/s41598-018-23590-1 PMC5876367

[CR57] Panda R, Bharath RD, Upadhyay N, Mangalore S, Chennu S, Rao SL (2016) Temporal dynamics of the default mode network characterize meditation-induced alterations in consciousness. Front Hum Neurosci 10:372 27499738 10.3389/fnhum.2016.00372 PMC4956663

[CR58] Pascarella A, Thölke P, Meunier D, O’byrne J, Lajnef T, Raffone A, Jerbi K (2025) Meditation induces shifts in neural oscillations, brain complexity, and critical dynamics: novel insights from MEG. Neurosci Conscious 2025(1):niaf047 41287816 10.1093/nc/niaf047 PMC12640546

[CR59] Pascual-Marqui RD, Michel CM, Lehmann D (1994) Low-resolution brain electromagnetic tomography (LORETA): a new method. Electroencephalogr Clin Neurophysiol 90:305–323

[CR60] Pascual-Marqui RD, Michel CM, Lehmann D (1995) Segmentation of brain electrical activity into microstates: model estimation and validation. IEEE Trans Biomed Eng 42(7):658–665. 10.1109/10.391164 7622149 10.1109/10.391164

[CR61] Patañjali. (1890). The Yoga-Sûtras of Patanjali (B. Dhar, Trans.). Project Gutenberg.

[CR62] Raichle ME, MacLeod AM, Snyder AZ, Powers WJ, Gusnard DA, Shulman GL (2001) A default mode of brain function. Proc Natl Acad Sci U S A 98(2):676–682 11209064 10.1073/pnas.98.2.676 PMC14647

[CR63] Seitzman BA, Abell M, Bartley SC, Erickson MA, Bolbecker AR, Hetrick WP (2017) Cognitive manipulation of brain electric microstates. Neuroimage 146:533–543. 10.1016/j.neuroimage.2016.10.002 27742598 10.1016/j.neuroimage.2016.10.002 PMC5321823

[CR64] Segal ZV, Williams JMG, Teasdale JD (2013) Mindfulness-based cognitive therapy for depression, 2nd edn. Guilford Press

[CR65] Tarailis P, Koenig T, Michel CM, Griškova-Bulanova I (2024) The functional aspects of resting EEG microstates: a systematic review. Brain Topogr 37(2):181–217. 10.1007/s10548-023-00958-9 37162601 10.1007/s10548-023-00958-9

[CR66] Tarailis P et al (2025) Self-related thought alterations associated with intrinsic brain dysfunction in mild cognitive impairment. Sci Rep 15:12279. 10.1038/s41598-025-97240-8 40210901 10.1038/s41598-025-97240-8 PMC11986127

[CR67] Vago DR, Zeidan F (2016) The brain on silent: mind wandering, mindful awareness, and states of mental tranquility. Ann N Y Acad Sci 1373(1):96–113 27398642 10.1111/nyas.13171 PMC5866730

[CR68] Wallace BA (1999) The Buddhist tradition of Samatha: Methods for refining and examining consciousness. In: Hameroff SR, Kaszniak A, Scott AC (eds) Toward a science of consciousness III. MIT Press, pp 143–154

[CR69] Zanesco AP (2024) Normative temporal dynamics of resting EEG microstates. Brain Topogr 37(2):243–264. 10.1007/s10548-023-01004-4 37702825 10.1007/s10548-023-01004-4

[CR70] Zanesco AP, Denkova E, Jha AP (2021a) Self-reported mind wandering and response time variability differentiate prestimulus electroencephalogram microstate dynamics during a sustained attention task. J Cogn Neurosci 33(1):28–45 33054554 10.1162/jocn_a_01636

[CR71] Zanesco AP, Skwara AC, King BG, Powers C, Wineberg K, Saron CD (2021b) Meditation training modulates brain electric microstates and felt states of awareness. Hum Brain Mapp 42(10):3228–3252 33783922 10.1002/hbm.25430 PMC8193519

[CR72] Zarka D, Cevallos C, Ruiz P, Petieau M, Cebolla AM, Bengoetxea A, Cheron G (2021) Trait and state mindfulness modulate EEG microstates. medRxiv Preprint. 10.1101/2021.11.22.21266675

[CR73] Zarka D, Cevallos C, Ruiz P, Petieau M, Cebolla AM, Bengoetxea A, Chéron G (2024) Electroencephalography microstates highlight specific mindfulness traits. Eur J Neurosci 59(7):1753–1769. 10.1111/ejn.16247 38221503 10.1111/ejn.16247

